# Evaluation of postnatal outcomes from a group antenatal care intervention in Nigeria: a quasi-experimental study

**DOI:** 10.7189/jogh.16.04023

**Published:** 2026-03-06

**Authors:** W Douglas Evans, Jeffrey B Bingenheimer, Taiseer Zaman, Samson Babatunde Adebayo, Fasiku Adekunle David, Sani Ali Gar, Masduk Abdulkarim

**Affiliations:** 1 The George Washington University, Milken Institute School of Public Health, Washington, DC, USA; 2 Data Research and Mapping Consults, Abuja, Nigeria; 3 Gates Foundation, Nigeria Country Office, Abuja, Nigeria

## Abstract

**Background:**

In previous studies in low- and middle-income countries (LMICs), group antenatal care (gANC) has been shown to increase health facility delivery and enhance the care experience for both women and providers. Here, we investigate whether those benefits lead to improvements in postnatal outcomes.

**Methods:**

We examine three time points of data on participation in the gANC programme as an independent variable to predict postnatal mother and newborn health outcomes using multivariate analysis and propensity matching techniques. This approach aimed to isolate the causal effect of the number of gANC meetings attended on the probability of postnatal healthcare checks for the mother and infant, modern contraception utilisation, and breastfeeding. To achieve this, we used inverse-probability weighting in addition to adjusted multivariate logistic regression models.

**Results:**

We observed high retention at final follow-up and high attendance overall, with higher attendance associated with a variety of background and sociodemographic variables. Participants’ attrition (under 24%) was well within power analysis assumptions. We found evidence of a strong positive relationship between greater gANC session attendance and mothers’ postnatal health checks, breastfeeding initiation, and modern contraception utilisation. Adjustment for a set of sociodemographic and prior pregnancy- and delivery-related variables *via* inverse probability weighting suggested that a positive effect on these outcomes generally persists, especially at the highest levels of gANC session attendance.

**Conclusions:**

The gANC programme at scale in Nigeria produced high levels of participation and resulted in positive postnatal outcomes among women who attended most of the meetings.

Group antenatal care (gANC) has been shown to be effective in increasing delivery in healthcare facilities in low- and middle-income countries (LMICs) and can enhance the care experience for both women and providers [1]. Nigeria is an LMIC with high antenatal care (ANC) non-utilisation rates, with its northern region having rates below 50% [2]. Implementation research is needed to understand how to promote gANC in real-world settings at scale in LMICs and to identify the full range of its potential benefits for mothers, newborns, and healthcare providers.

Previous research has found that financial, access, and other ability barriers limit ANC in LMIC [3]. A recent study showed how gANC can enhance healthcare experiences during pregnancy including social support, peer learning, active health participation, health education, and satisfaction or engagement with care [4]. Additionally, shared health activities and women-led, group-based discussions have been shown to improve relationships between women and midwives [5,6].

One critical area where gANC may be beneficial is in improving maternal knowledge, attitudes, beliefs, and health behaviours in the postpartum period. The programme provides support and information on behaviours such as the importance of health checks for mother and newborn, breastfeeding, family planning, and vaccination [7]. One gANC study found that women in the intervention group had significantly higher knowledge of newborn danger signs and healthy newborn practices over time compared to those receiving individual antenatal care (iANC) [8].

While an important focus of gANC is on increasing healthcare facility utilisation, with previous studies showing it is effective in achieving this outcome [9], the programme also includes important postnatal health content. Lanyo *et al*. [10] conducted a scoping review to identify positive impacts of gANC on the adolescent population and the unique and context-specific challenges hindering access to and use of gANC in LMICs. They found that gANC leads to better adherence to care, increased empowerment through knowledge, enhanced social support, and improved newborn health quality.

Previous studies have demonstrated the positive effects of gANC on birth preparedness and complication readiness (BPCR) and their effect on maternal and neonatal outcomes [11]. Low BPCR contributes to high maternal and neonatal morbidity and mortality in LMICs. Kukula *et al*. [11] found that BPCR scores significantly increased in the gANC group compared to the iANC group.

There is evidence that gANC can affect other postnatal outcomes, as well. In a study in India, women who participated in gANC were also more likely to take prenatal vitamins and iron, use contraception at two months postpartum, and report higher satisfaction with the information provided, their relationship with healthcare providers, and overall quality of care [12]. A cluster-randomised controlled trial in Ghana comparing gANC with standard care found that, by 34 weeks of gestation, women in the intervention group had significantly greater knowledge of modern contraception (MC) methods and a stronger intention to use them after childbirth [13]. The same trial also reported higher MC uptake in the intervention group at most postnatal time points.

Overall, gANC presents a promising alternative to conventional ANC by integrating education, peer support, and active health engagement. Its ability to improve maternal knowledge, preparedness for birth, postnatal care utilisation, and the uptake of key maternal health behaviours highlights its potential to transform ANC service delivery, particularly in resource-limited settings.

In this study, we report on the third and final follow-up (endline) evaluation of a large-scale gANC implementation project in Kaduna and Kano states in northern Nigeria. These areas are priorities for interventions to improve maternal and child health outcomes and increase ANC utilisation and care, based on recommendations from the Nigerian government and the Gates Foundation [14]. The overarching project, of which this study is the final part, is the first large-scale, quasi-experimental, longitudinal evaluation of gANC in Nigeria. Endline findings fill a gap in knowledge about the postnatal outcomes of real-world implementation and effectiveness in these settings. The baseline phase of the study was implemented in Kaduna and Kano states in Nigeria in March and April 2024 [15]. The midline phase – follow-up data collection from the longitudinal cohort of women recruited at baseline – was implemented in Kaduna and Kano in October and November 2024 and found that higher levels of gANC participation were associated with higher healthcare facility delivery [9].

Here, we report on an analysis of the follow-up and final endline data from the third phase of the project, collected in July 2025. Our key research questions focus on the implementation and primary effectiveness of gANC in promoting positive postnatal outcomes for the mother and newborn. Specifically, we asked: To what extent did gANC participation increase postnatal maternal health checks? To what extent did gANC participation increase child health checks within two months of birth? To what extent did gANC participation increase breastfeeding initiation within one hour of birth? To what extent gANC participation increase MC use within six months of birth?

## METHODS

### Study design

The overall project adopted an observational, prospective design with three waves of data collection at eight month intervals: baseline, when pregnant women were initially sampled, recruited, and interviewed for the first time (March and April, 2024); midline, in which participants were interviewed again (November 2024); and endline, when participants were interviewed for a third time (June and July, 2025) (Checklist S1 in the **Online Supplementary Document**). Details of the sampling, recruitment, and data collection methods have been described in detail elsewhere [16]. Briefly, we sampled two local government areas (LGA) from each of the three senatorial districts in two Nigerian States: Kaduna and Kano. Next, we randomly sampled eight health facilities from each sampled LGA. Finally, we sampled 25 women aged 15–49 years who were pregnant and participating in or planning to participate in gANC. Some deviations from this plan occurred due to the security situation in parts of each state and due to the small number of health facilities in some LGAs. Overall, however, 2469 participants were recruited, consented, enrolled in the study, and completed baseline interviews. Field teams collected data *via* interviews in and around participants’ homes using questionnaires programmed into tablet computers.

### Intervention

The focal intervention is gANC, which has been described in detail elsewhere [15]. It consists of five facilitator-led meetings with small groups of pregnant women, each lasting approximately one hour and carried out at the health facility at which the facilitator is stationed. The first meeting is intended for women at 20–24 weeks of gestational age and focuses on orienting participants to the group and preventing problems during pregnancy. The second meeting, intended for women at 24–28 weeks of gestational age, attempts to recognised and respond to problems women encountered during pregnancy. Participants’ male partners are invited to the third meeting, which is intended for women at 28–32 weeks of gestational age and focuses on birth preparedness, complication readiness, and post-partum family planning. The fourth session is intended for women at 32–36 weeks of gestational age and covers signs of labour, preventing problems after birth, and post-partum family planning intentions. The final session, for women at 36–40 weeks of gestational age, includes final birth planning, breastfeeding, and recognising and responding to maternal and infant health problems in the post-partum period. All sessions include some medical services, such as blood pressure measurement to detect hypertension, detection and treatment of other pregnancy-related conditions, and provision of folic acid and micronutrient supplements. The gANC model is hypothesised not only to be less expensive to deliver per-person than traditional iANC, but also to be more effective by promoting information sharing and other forms of social support among participants. Our research team was not involved in any aspect of the gANC intervention, which was instead delivered by the health facilities from which study participants were recruited.

### Measures

The focal independent variable was the number of gANC meetings attended, assessed in midline interviews, by which time virtually all index pregnancies had ended, mostly in live births. Participants were asked, ‘Which group meetings did you attend?’, and the interviewer recorded whether the participant indicated that she attended each of the five gANC meetings, and if she did, asked a series of follow-up questions about services delivered at that meeting. From five variables in the resulting dataset, we obtained a count of the number of gANC meetings attended, which could range from zero (if the participant attended none) to five (if she attended all five).

We previously reported on the primary endpoint of facility delivery which was also assessed at midline [9]. Here, we focus on four secondary endpoints, all of which were assessed during endline interviews, and are operationalised insofar as possible to align with indicators for the Sustainable Development Goals, such as 3.1 (to reduce maternal mortality) and 3.2 (to reduce newborn mortality), and with large-scale studies assessing levels and trends of maternal and child health outcomes, such as the Demographic and Health Surveys and Multiple Indicator Cluster Surveys. The first of these is whether or not the mother received a post-natal health check within two weeks following delivery. To assess this variable, interviewers asked participants, ‘Did anyone check on your health while you were still in the facility?’ and, if they answered affirmatively, further asked them, ‘How long after delivery did the first check take place?’. For the latter, responses were recorded as a number and a unit (hours, days, or weeks). Based on the resulting variables, we created a binary indicator of whether the mother reported receiving a health check within one week following delivery.

Our second outcome variable, initiation of modern contraceptive use within six months of delivery, participants were asked, ‘Are you or your partner currently doing something or using any method to delay or avoid getting pregnant?’ Those who answered affirmatively were then asked about which method(s) they were using, and how long after delivery they started using them. Based on the resulting variables, we created a binary indicator of whether or not the participant initiated use of a modern contraceptive method (defined, consistent with other surveys, as female or male sterilisation, intrauterine device, injectables, implants, contraceptive pills, male or female condom, emergency contraception, the standard days method, or the lactational amenorrhea method) within six months following delivery.

Our third outcome was initiation of breastfeeding within one hour following delivery. To assess this variable, interviewers asked participants, ‘Did you ever breastfeed (baby name), or feed him/her your pumped/collected breastmilk?’ Those who answered affirmatively were then asked, ‘How long after birth did you first put (baby name) to the breast?’ We created a binary indicator of initiation of breastfeeding within one hour of delivery based on responses to these questions (and an analogous set of questions that were posed to participants who reported giving birth to twins).

Our fourth and final outcome was whether or not the baby (or babies, in the case of twin births) received a health check within the first two months following delivery. This variable was only assessed for participants who responded affirmatively to the question, ‘Is your most recent baby alive?’. A pair of analogous questions were posed to participants who reported twin births. Participants who reported that their baby (or at least one twin) was still alive were asked, ‘In the two months after (baby name) was born, did any healthcare provider or a traditional birth attendant check on his/her health?’ We created a binary indicator based on responses to the latter question, and omitted from analyses of this outcome participants whose baby (or both of whose twin babies) had died.

We also used several sociodemographic variables and variables characterising experiences with prior pregnancies and deliveries, all of which were assessed at baseline. The sociodemographic variables included age, education, employment, state and type of area (urban or rural) of residence, whether the participant had ever given birth before, and the number of living children. We also assessed religion and marital status; however, as almost all participants were married and Muslim, we did not use them in the analysis. Prior pregnancy and delivery experience variables included whether the participant had experienced any problems during a prior pregnancy or delivery, whether their most recent delivery occurred at a health facility, whether a skilled birth attendant was present at their most recent delivery, whether the participant received a health check within one week of their most recent prior delivery, and whether they attended any postnatal health visit following their most recent prior delivery. We summarised these prior pregnancy and delivery variables into a risk index ranging from zero to five, with higher scores indicating greater risk, based on previous research [17]. We use these variables to characterise the sample and to control for confounding in our primary analyses.

### Data analysis

Our analysis focused on participants who were located and reinterviewed at both midline and endline and who reported that their index pregnancy had ended in a live birth. For our fourth outcome variable (postnatal health check for infant within two months following delivery), we further limited our analysis to participants who reported at endline that their baby was still alive. After merging the baseline, midline, and endline datasets, we first examined levels, reasons for, and patterns of attrition between waves. We then used frequencies and descriptive statistics to characterise the analytic sample in terms of baseline sociodemographic and prior pregnancy and delivery related variables, as well as our focal independent and dependent variables. Next, we examined the extent to which baseline sociodemographic and prior pregnancy and delivery related variables were associated with number of gANC sessions attended. We did this by obtaining means (x̄) and standard deviations (SDs) of the number of sessions attended within strata defined by the background variables, and by using linear regression models predicting the number of sessions in relation to each of the background variables. These analyses provided preliminary insight into the first condition of confounding: whether and how potential confounding variables are associated with the exposure of interest.

We then examined the extent to which the same baseline sociodemographic and prior pregnancy and delivery related variables were associated with each of our four outcome variables. We did this by cross-tabulating those outcome variables with each of the background variables to determine the percentage of participants reporting each outcome within each stratum of each background variables, and by using logistic regression models to determine the statistical significance of those associations. These analyses provide an initial determination of whether and how the second condition of confounding, that a confounder influences the outcome of interest, was satisfied.

Our primary analysis, intended to isolate the cause-and-effect relationship between the number of gANC meetings attended and our four outcome variables, was inverse-probability weighting (IPW) [14]. This analytic approach is similar to propensity score analysis in that potential confounders are used to model the probability that each participant experiences each level of the exposure variable. Rather than matching or stratifying on the resulting predicted probabilities, however, this approach uses the inverse of each participants’ predicted probability of experiencing the observed level of the exposure variable as a weighting variable in subsequent analyses linking that exposure to the outcome or outcomes of interest. Whereas propensity-score matching and stratification approaches are most readily applied to dichotomous exposure variables, the IPW approach easily generalises to situations where, as here, the exposure of interest has more than two levels.

We conducted all analyses in Stata, version 19.0 (StataCorp LLC, College Station, Texas, USA), and specifically used the ‘teffects ipwra’ command, which utilises a multinomial logistic regression model, to relate the focal exposure variable to the potential confounders and to obtain the IPWs. It is doubly robust in that the potential confounders are then used again as covariates in IPW regression models linking the exposure of interest to each outcome. For all analyses involving statistical inference, we obtained clustered rather than conventional standard errors and *P*-values using LGA as the clustering variable using Stata’s vce() option, as in our previous study [9].

## RESULTS

Of the original sample of 2469 pregnant women, 1878 (76.1%) were located and reinterviewed at the endline follow-up, which is a higher retention rate that required in our sampling plan. The sample was close to evenly distributed between the two study states of Kaduna (46.2%) and Kano (53.8%), and is heterogeneous with respect to age, education, employment status, and number of living children. A higher proportion of participants resided in rural areas than in urban areas, and over 76% had given birth before. With respect to prior pregnancy experiences, just over one-quarter (26.6%) had experienced problems in their most recent prior pregnancy, and approximately 60% had delivered their most recent pregnancy in a health facility as opposed to at home. Most prior deliveries involved the presence of a skilled birth attendant, although a substantial minority occurred without one. Over 86% reported having had a postnatal health check within one week of their most recent delivery, and about 89% had at least one postnatal care appointment following that delivery. Just over 60% had one or more prior pregnancy risk factors (≥1 on the scale) and almost 35% had two or more risk factors (**Table 1**).

**Table 1:** Distribution of sociodemographic and prior pregnancy- and delivery-related variables among participants in the analytic sample (n = 1878)

	n (%)
**State**	
Kaduna	867 (46.2)
Kano	1011 (53.8)
**Age group in years**	
15–19	237 (12.6)
20–24	677 (36.0)
25–29	486 (25.9)
30–34	289 (15.4)
≥35	189 (10.1)
**Education**	
Never attended formal school	470 (25.0)
Primary	394 (21.0)
Secondary	682 (36.3)
Higher	199 (10.6)
Qur’anic/Islamiyya	127 (6.8)
Other	6 (0.3)
**Employment status**	
Unemployed	781 (41.6)
Employed	104 (5.5)
Own a business	993 (52.9)
**Residence**	
Urban	695 (37.0)
Rural	1183 (63.0)
**Ever given birth before**	
No	443 (23.6)
Yes	1435 (76.4)
**Number of living children**	
0	473 (25.2)
1	358 (19.1)
2	281 (15.0)
3	264 (14.1)
4	207 (11.0)
≥5	295 (15.7)
**Experienced problems during prior pregnancy/delivery**	
No or no prior pregnancy/delivery	1379 (73.4)
Yes	499 (26.6)
**Place of delivery for last pregnancy**	
Facility delivery or no prior pregnancy/delivery	1131 (60.2)
Home or other	747 (39.8)
**Skilled birth attended at last delivery**	
Yes or no prior pregnancy/delivery	1353 (72.0)
No	525 (28.0)
**Postnatal health check within one week of last delivery**	
Yes or no prior pregnancy/delivery	1619 (86.2)
No	259 (13.8)
**Any postnatal care appointments after last delivery**	
Yes or no prior pregnancy/delivery	1669 (88.9)
No	209 (11.1)
**Prior pregnancy risk index**	
0	744 (39.6)
1	480 (25.6)
2	338 (18.0)
3	196 (10.4)
4	105 (5.6)
5	15 (0.8)

The sub-sample of 1739 mothers who had a live birth was about evenly split between those who attended four or five meetings (49.7%) and those who attended three or fewer. Among this sub-sample, just over half (51.3%) had a postnatal health check within one week of delivery. About 31% used MC within six months of delivery, over half (53.5%) initiated breastfeeding within one hour after delivery, and three-quarters had a health check for their newborn within two months of birth (**Table 2**).

**Table 2:** Distribution of group antenatal care meeting attendance and postnatal health outcomes (n = 1739)

	n (%)
**Number of gANC meetings attended during index pregnancy**	
0	181 (9.6)
1	166 (8.8)
2	247 (13.2)
3	350 (18.6)
4	370 (19.7)
5	564 (30.0)
**Postnatal health check for mother within two days of delivery**	
No	915 (48.7)
Yes	963 (51.3)
**Modern contraceptive use within six months of delivery**	
No	1298 (69.1)
Yes	580 (30.9)
**Breastfeeding initiated within one hour of delivery**	
No	874 (46.5)
Yes	1004 (53.5)
Postnatal health check for newborn within two months of birth*	
No	433 (24.9)
Yes	1306 (75.1)

gANC – group antenatal care

*The questionnaire item about postnatal health check for newborn was not administered to participants who said that their baby had died, so the sample size for this outcome was 1739.

The number of meetings attended was higher in Kaduna (x̄ = 3.71, SD = 1.40) than in Kano (x̄ = 2.62, SD = 1.72) (*P* = 0.013). None of the other variables were significantly associated with number of meetings attended (**Table 3**).

**Table 3:** Associations between background variables and number of group antenatal care meetings attended (n = 1878)

	Mean (SD)	*P*-value
**State**		0.013
Kaduna	3.71 (1.40)	
Kano	2.76 (1.72)	
**Age group in years**		0.415
15–19	3.01 (1.69)	
20–24	3.22 (1.65)	
25–29	3.34 (1.62)	
30–34	3.10 (1.68)	
≥35	3.17 (1.59)	
**Education**		0.294
Never attended formal school	2.94 (1.68)	
Primary	3.17 (1.65)	
Secondary	3.28 (1.64)	
Higher	3.64 (1.51)	
Qur’anic/Islamiyya	3.09 (1.62)	
Other	3.67 (1.21)	
**Employment**		0.088
Unemployed	3.02 (1.71)	
Employed	3.75 (1.47)	
Own a business	3.28 (1.59)	
**Residence**		0.624
Urban	3.33 (1.59)	
Rural	3.13 (1.67)	
**Ever given birth before**		0.977
Yes	3.20 (1.67)	
No	3.20 (1.64)	
**Number of living children**		0.337
0	3.19 (1.68)	
1	3.22 (1.67)	
2	3.28 (1.71)	
3	3.34 (1.59)	
4	3.08 (1.66)	
≥5	3.07 (1.55)	
**Prior pregnancy risk index**		0.235
0	3.37 (1.58)	
1	3.26 (1.60)	
2	3.13 (1.71)	
3	2.85 (1.70)	
4	2.75 (1.75)	
5	2.00 (1.69)	

SD – standard deviation, x̄ – mean

In the subsample of women who delivered their baby in a health facility (n = 1739), the rates of mothers’ postnatal health checks, breastfeeding initiation, and MC use were all higher in Kaduna than in Kano. There was no significant difference in child postnatal health checks between the two states. There are variable levels of MC use and breastfeeding initiation by age, with women in the 20–29 year range generally having higher levels of these outcomes. Having higher education was associated with higher rates of mothers’ postnatal health checks, breastfeeding initiation, and MC use, but not for child postnatal health checks. Being employed was associated with MC use and breastfeeding initiation. Living in an urban setting was associated with higher rates of mothers’ postnatal health checks, breastfeeding initiation, and MC use. Having given birth previously was associated with higher levels of mothers’ postnatal health checks. There were variable levels of associations between having one or more children (compared to none) and rates of mothers’ postnatal health checks, breastfeeding initiation, and MC use. Finally, a lower prior pregnancy risk index was associated with rates of mothers’ postnatal health checks, MC use, and child postnatal health checks (Table S1 in the **Online Supplementary Document**).

Overall, in the adjusted models, attending four or more meetings was associated with higher rates of mothers’ postnatal health checks, and attending three or more in the IPW adjusted models. MC use within six months was higher with two or more meetings attended in the unadjusted and adjusted models, but only with five or more in the IPW adjusted models. Initiation of breastfeeding within one hour of birth was associated with five meetings in the unadjusted and adjusted models. Surprisingly, getting a postnatal health check for the newborn was generally lower with three or more meetings attended (with the exception of four meetings in the unadjusted model) (Table S2 in the **Online Supplementary Document**).

## DISCUSSION

In this study, we examined gANC implementation in our longitudinal sample across three waves of data collection, and the effects of participation on two postnatal outcomes: the implementation of gANC intervention and longitudinal follow-up of women who participated in gANC, and whether attending a larger number of gANC sessions increased the likelihood of four key postnatal health outcomes (mothers’ postnatal health checks, postpartum MC use within six months, breastfeeding initiation within one hour of birth, and child postnatal health checks).

In terms of study implementation and follow-up, we found that there was high retention at endline (76.1%), and generally high gANC meeting attendance, especially in Kaduna (x̄ = 3.71). These endline findings confirm the results from our midline study, which concluded that successful implementation and high retention were significantly successful in low resource settings such as Kaduna and Kano [9]. Also as in our midline study, participation rates skewed to the high end of the scale of possible meetings attended, with nearly half (49.7) of participants attending four or five meetings. The gANC was successfully implemented at scale with low attrition in Kaduna and Kano, consistent with prior research [1].

In terms of our key research questions at endline, we note that higher levels of participation in gANC are associated with more positive on postnatal maternal and newborn outcomes. In terms of causal inference, there was evidence of a strong positive relationship between greater gANC session attendance and mothers’ postnatal health checks, breastfeeding initiation, and MC utilisation. Adjustment for a set of socio-demographic and prior pregnancy- and delivery-related variables *via* IPW suggested that a positive effect on these outcomes generally persists, especially at the highest levels of gANC session attendance (four or five sessions).

However, we found that there was a negative relationship between gANC session attendance and child health checks within two months of birth. While this is an unwelcome finding, there are some possible explanations for these results. In separate qualitative interviews with participants, some women reported that when they took their infants for vaccinations and returned with the infants having swollen arms and crying. Consequently, their husbands stopped them from taking the infants back to the health facility for health checks. As noted in the theory of change for this campaign, reported previously, external factors such as family, community, and social norms about healthcare utilisation, family planning, and vaccinations can negatively influence child health check utilisation [18]. Other explanations, such as a failure of the intervention to effectively address the importance of child health checks are possible, and should be investigated in future research.

Overall, our add to and extend previous studies of gANC [19]. In addition to improving facility delivery, gANC participation improves many postnatal health outcomes for the mother and newborn. The programme can help promote maternal and child health in LMICs and may be scalable in LMICs [20].

### Limitations

Some limitations of this study should be noted, two of which are similar to those of our midline study. First, both the focal independent variable of number of gANC meetings attended, as well as the dependent variable of facility delivery of the index pregnancy, were assessed *via* participant self-reporting. This could lead to upward or downward bias in our observed associations between gANC meeting attendance and facility delivery. Both gANC meeting attendance and facility delivery may be subject to some degree of social desirability bias, and some participants may be more inclined than others to provide socially desirable responses. It is possible that social desirability bias may have inflated self-reports of positive postpartum health behaviours.

The second main limitation is the possibility of omitted variable bias. While we had a substantial set of covariates that included both sociodemographic background variables and characteristics of participants’ previous pregnancy, delivery, and postpartum experiences, other confounders could have been omitted from this set. This concern may be mitigated to some extent by the associations between included covariates and omitted confounders. One possible confounder that was not included in our analysis, for example, was the distance between each participant’s dwelling and the health facility, or more generally, the difficulty involved in traveling from the dwelling to the facility. Such omitted variable bias may have affected the results of the IPW analyses.

Third, and specific to this endline study, the negative effects of participation on child health checks may also have to do with omitted variables, namely family, community, and other normative influences. We did not measure the husbands’ roles in maternal and newborn healthcare, and based on anecdotal information, there is reason to believe that such factors may have contributed to the negative findings on child health checks. Other explanations, such as a failure of the intervention to effectively address child health checks, should also be investigated.

Finally, attendance in the gANC programme may be driven by women who are more health-motivated, healthier, or better supported are both more likely to attend more sessions and more likely to engage in postnatal care behaviours. Attendance is itself an outcome of complex social and structural factors, and there is potential for reverse-causality in the analysis. Future studies should account for this potential confounding factor.

### Future research

This is the third and final study in a series of longitudinal studies on the gANC program in Nigeria. Future research should examine methods to optimise gANC, such as isolating specific services that are most engaging, utilised, and efficacious in promoting maternal and child health behaviours [21]. Family, community, and normative influences on maternal and newborn healthcare should also be examined. Consistency of intervention delivery across sites is important, and heterogeneity of implementation should be studied in future studies. Additional outcomes not included in this study, specifically vaccination and other long-term postnatal outcomes of gANC participation, should be evaluated. The promotion of gANC through channels such as telehealth and social media or patient-provider apps, which could mitigate factors such as the inability to travel to a healthcare facility, should be studied. These approaches have potential to increase programme participation and improve both pre- and postnatal outcomes [21].

## CONCLUSIONS

Implementation of the gANC program at scale in Nigeria suggested that participation that the program improved postnatal maternal health check, breastfeeding, and family planning outcomes. The program is an evidence-based approach to promoting maternal and child healthcare in LMICs. Future studies should examine long-term effects of the program on participants and ways to optimise gANC implementation.

## Additional material


Online Supplementary Document
