# Supplementary material for: Evaluation of postnatal outcomes from a group antenatal care intervention in Nigeria: a quasi-experimental study
Source: J Glob Health. 2026 Mar 6;16:04023. doi: 10.7189/jogh.16.04023 (PMC12964324; doi:10.7189/jogh.16.04023)
Supplement: Online Supplementary Document [file jogh-16-04023-s001.pdf]

Supplement to: Evans WD, Bingenheimer JB, Zaman T, Adebayo SB, David FA, Gar SA. Evaluation of postnatal outcomes from a group antenatal care intervention in Nigeria: a quasi-experimental study. J Glob Health. 2026;16:04023.

Table S1. Associations between Background Variables and Percent Delivering in a Health Facility (n=1,739)*

	Postnatal Health Check for Mother within 2 Days		Post-Partum MC Use within 6 Months		Breastfeeding within One Hour of Birth		Postnatal Health Check for Baby within 2 Months	
	Percent	p-value	Percent	p-value	Percent	p-value	Percent	p-value
State		0.019		0.131		0.000		0.640
Kaduna	66.3		36.6		71.2		73.2	
Kano	38.4		26.0		38.3		76.7	
Age Group		0.515		0.000		0.000		0.338
15-19	45.6		23.2		45.6		72.9	
20-24	52.7		32.1		53.5		76.6	
25-29	53.1		34.2		61.3		74.3	
30-34	50.5		33.6		50.9		72.1	
35+	49.7		23.8		47.1		79.2	
Education		0.000		0.000		0.000		0.279
Never attended formal school	31.3		20.0		37.0		76.0	
Primary	43.4		28.7		49.5		76.4	
Secondary	63.9		34.9		61.6		76.1	
Higher	81.9		50.8		73.4		67.4	
Qur'anic/Islamiyya	33.9		25.2		52.8		74.8	
Other	50.0		33.3		33.3		75.0	
Employment		0.130		0.000		0.027		0.367
Unemployed	48.0		28.9		49.6		75.8	

Employed	66.4		46.2		72.1		70.4	
Own a business	52.3		30.8		54.6		75.1	
Residence		0.000		0.015		0.000		0.102
Urban	78.4		39.0		72.1		68.3	
Rural	35.3		26.1		42.5		79.1	
Ever Given Birth Before		0.001		0.616		0.703		0.738
Yes	61.4		31.8		54.4		74.4	
No	48.2		30.6		53.12		75.3	
Number of Living Children		0.000		0.000		0.000		0.119
0	60.7		31.7		54.6		75.1	
1	55.3		36.3		57.8		73.7	
2	47.7		32.0		54.1		78.9	
3	53.0		28.8		59.1		75.3	
4	42.5		31.4		53.1		78.2	
5+	39.3		23.4		41.0		70.8	
Prior Pregnancy Risk Index		0.000		0.061		0.000		0.038
0	64.3		35.1		60.6		73.2	
1	50.8		32.1		52.5		75.1	
2	36.7		26.0		51.2		74.2	
3	37.8		20.9		41.3		81.1	
4	38.1		31.4		40.0		82.5	
5	20.0		20.0		33.3		64.3	

MC – modern contraception, n – number.

*The questionnaire item about the postnatal health check for the newborn was not administered to participants who reported that their baby had died; therefore, the sample size for this specific outcome is n = 1739

Table S2. Unadjusted and Adjusted Associations between Number of gANC Meetings Attended and Four Outcomes (n=1878, 1857, 1739, 1721) †

		**Unadjusted**		**Regression Adjusted**		**IPW Adjusted**	
**A. Health Check for Mother within 2 Days**							
gANC Meetings	**%**	**OR**	**(95% C.I.)**	**AOR**	**(95% C.I.)**	**AOR**	**(95% C.I.)**
0	34.8	0.70	(0.40-1.22)	0.91	(0.49-1.70)	1.05	(0.90-1.20)
1	43.4	1.00	(reference)	1.00	(reference)	1.00	(reference)
2	41.7	0.93	(0.60-1.45)	1.08	(0.67-1.73)	1.02	(0.92-1.11)
3	51.1	1.37	(0.80-2.33)	1.44	(0.83-2.50)	1.09	(1.00-1.19)*
4	55.7	1.64	(0.90-2.97)	1.78	(1.11-2.84)*	1.13	(1.03-1.23)**
5	60.3	1.98	(0.98-4.00)	1.93	(1.15-3.26)*	1.16	(1.06-1.26)**
**B. Post-partum MC Use within 6 Months**							
gANC Meetings	**%**	**OR**	**(95% C.I.)**	**AOR**	**(95% C.I.)**	**AOR**	**(95% C.I.)**
0	21.0	0.87	(0.47-1.59)	0.95	(0.50-1.81)	1.06	(0.92-1.20)
1	23.5	1.00	(reference)	1.00	(reference)	1.00	(reference)
2	31.2	1.47	(1.11-1.96)**	1.60	(1.19-2.15)*	1.07	(0.97-1.17)
3	31.1	1.47	(1.07-2.02)*	1.48	(1.05-2.07)*	1.08	(0.99-1.17)
4	31.1	1.47	(1.06-2.04)*	1.44	(1.01-2.07)*	1.07	(0.98-1.15)
5	35.8	1.82	(1.30-2.54)***	1.65	(1.25-2.18)***	1.09	(1.00-1.18)*
**C. Immediate Initiation of Breastfeeding**							
gANC Meetings	**%**	**OR**	**(95% C.I.)**	**AOR**	**(95% C.I.)**	**AOR**	**(95% C.I.)**

0	42.0	0.82	(0.50-1.32)	1.20	(0.84-1.70)	1.06	(0.92-1.21)
1	47.0	1.00	(reference)	1.00	(reference)	1.00	(reference)
2	47.0	1.00	(0.64-1.57)	1.17	(0.70-1.94)	1.03	(0.93-1.13)
3	51.7	1.21	(0.85-1.72)	1.12	(0.84-1.48)	1.01	(0.92-1.10)
4	54.6	1.36	(0.95-1.93)	1.25	(0.99-1.57)	1.03	(0.94-1.12)
5	62.2	1.86	(1.13-3.07)*	1.45	(1.16-1.79)**	1.06	(0.97-1.15)
**D. Health Check for Baby within 2 Months**							
gANC Meetings	**%**	**OR**	**(95% C.I.)**	**AOR**	**(95% C.I.)**	**AOR**	**(95% C.I.)**
0	74.6	0.64	(0.26-1.58)	0.58	(0.24-1.39)	0.94	(0.85-1.03)
1	82.1	1.00	(reference)	1.00	(reference)	1.00	(reference)
2	83.7	1.12	(0.58-2.17)	1.07	(0.54-2.10)	0.95	(0.87-1.02)
3	73.5	0.61	(0.40-0.92)*	0.59	(0.38-0.91)*	0.90	(0.84-0.96)**
4	76.2	0.70	(0.44-1.11)	0.66	(0.45-0.98)*	0.91	(0.85-0.97)**
5	69.7	0.50	(0.30-0.84)**	0.46	(0.32-0.67)***	0.84	(0.79-0.90)***

AOR – adjusted odds ratio, CI – confidence interval, gANC – group antenatal care, IPW – inverse-probability weighting, n – number, OR – odds ratio.

*p < 0.05, **p < 0.01, ***p < 0.001.

†Twenty-one participants were excluded from the inverse-probability weighted (IPW) analysis for the first three outcomes and 18 for the fourth outcome due to extreme treatment probabilities violating the overlapping support assumption. This reduced the IPW analytic sample from n = 1878 to n = 1857 for the first three variables and from n = 1739 to n = 1721 for the fourth endpoint.
